# T208. LONGITUDINAL FEASIBILITY AND ACCEPTABILITY OF THE EXPRESS SMARTPHONE APP: RECRUITMENT, RETENTION AND PRELIMINARY FINDINGS

**DOI:** 10.1093/schbul/sby016.484

**Published:** 2018-04-01

**Authors:** Emily Eisner, Sandra Bucci, Richard Emsley, Christine Barrowclough, Richard Drake

**Affiliations:** University of Manchester

## Abstract

**Background:**

Relapse of schizophrenia is common, has profound, adverse consequences for patients and is costly to health services. Early signs interventions aim to use warning signs of deterioration to prevent full relapse. These interventions show promise but could be further developed. There is preliminary evidence that adding ‘basic symptoms’ to conventional early signs of relapse may improve relapse prediction. Basic symptoms are subtle, sub-clinical disturbances in one’s experience of oneself and the world that can include, for example, perceptual changes, mild subjective cognitive problems and decreased tolerance of stressors. This study aimed to evaluate the feasibility and acceptability of using the ExPRESS smartphone app to monitor both conventional early signs and basic symptoms as possible predictors of relapse.

**Methods:**

Patients who had experienced a relapse of schizophrenia within the past year took part in a screening interview. Those with at least one basic symptom emerging prior to a previous relapse were eligible for the longitudinal feasibility study. Consenting participants were asked to use the ExPRESS smartphone app once a week for 6 months, answering questions on their experience of conventional early signs, basic symptoms and psychotic symptoms. When app responses indicated an increase in psychotic symptoms above a pre-defined threshold, the researcher conducted the PANSS positive symptoms interview over the phone to assess whether the symptom increase was indicative of relapse. At the end of the follow-up period, face-to-face qualitative interviews were conducted to explore participants’ experiences of using the phone app and reasons for study dropout.

**Results:**

82% (18/22) of those screened were eligible for the longitudinal feasibility study and consented to participate. Of these, 72% (13/18) completed at least half of the weekly phone app assessments, with two participants dropping out of the study without completing any assessments on the phone app. Two participants met pre-defined relapse criteria during the 6 month follow up period. Initial findings from sixteen qualitative interviews are discussed, including interviews with the two participants who met relapse criteria and two study drop-outs.

**Discussion:**

The rate of recruitment to the longitudinal study was much higher than expected, since a higher than expected proportion of screened participants had experienced basic symptoms prior to a previous relapse. Retention rates were as expected, suggesting that the ExPRESS app is acceptable to patients with psychosis. Qualitative feedback from participants supports this conclusion. The results from this longitudinal feasibility study will inform the design of a well-powered definitive study prospectively examining the sensitivity and specificity of basic symptoms in predicting relapses of schizophrenia.

